# Supplementary material for: Diet-Induced Obesity in Mice Affects the Maternal Gut Microbiota and Immune Response in Mid-Pregnancy
Source: Int J Mol Sci. 2024 Aug 21;25(16):9076. doi: 10.3390/ijms25169076 (PMC11354285; doi:10.3390/ijms25169076)
Supplement: Supplementary file 1 [file ijms-25-09076-s001.zip › ijms-3139391-supplementary.pdf]

“**Diet-induced Obesity in Mice Affects the Maternal Gut Microbiota and Immune Response in Mid-pregnancy**” by L. Wekema, S. Schoenmakers, N. Schenkelaars, A. Laskewitz, R.H. Huurman, L. Liu, L. Walters, H.J.M. Harmsen, R.P.M. Steegers-Theunissen and M.M. Faas

Supplementary Materials

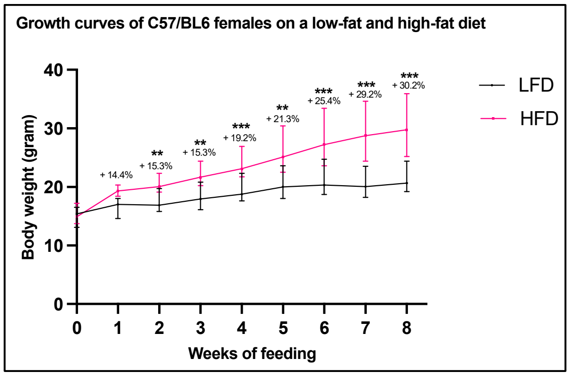

Supplementary Figure S1: Growth curves of female C57BL/6 mice fed a high-fat diet (HFD) (n = 8) or low-fat diet (LFD) (n = 8). Data are presented as median ± range. Per week the percentage of weight gain of the HFD mice is depicted as compared to the LFD mice. After 8 weeks of feeding, the body weight of the mice on the HFD (30.50 gram ± 1.59) exceeded the body weight of the mice on the LFD (21.28 gram ± 0.66) by > 30%. Mann-Whitney-U-test. ** $p < 0.01$, *** $p < 0.001$.

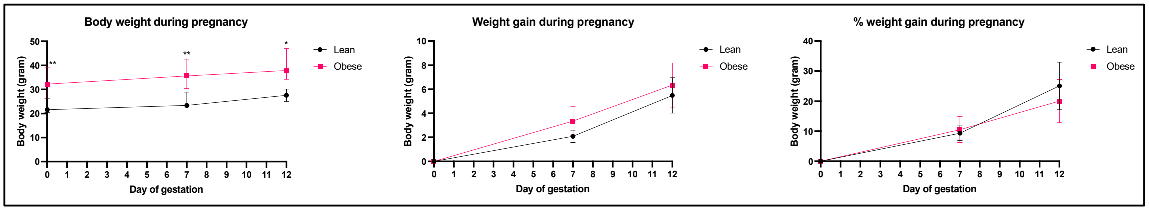

Supplementary Figure S2: Left: Body weight of lean (n = 8) and obese (n = 8) C57BL/6 mice during pregnancy at day 0, 7 and 12. Data are presented as median ± range. Kruskal-Wallis-test followed by Dunn’s multiple comparisons test. * $p < 0.05$, ** $p < 0.01$. During pregnancy the weight of the obese group remained significantly higher than the weight of the lean group at all days tested. Middle: Weight gain of lean (n = 8) and obese (n = 8) C57BL/6 mice during pregnancy at day 7 and 12 as compared to day 0. Data are presented as median ± range. Kruskal-Wallis-test followed by Dunn’s multiple comparisons test. Right: Percentage weight gain of lean (n = 8) and obese (n = 8) C57BL/6 mice during pregnancy at day 7 and 12 as compared to day 0. Data are presented as median ± range. Kruskal-Wallis-test followed by Dunn’s multiple comparisons test.

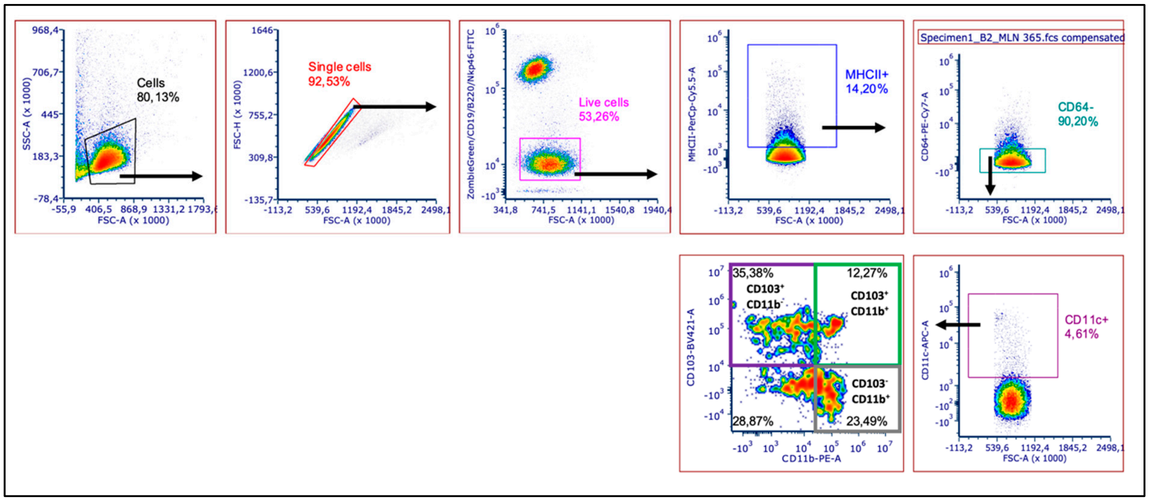

Supplementary Figure S3: Gating strategy for the determination of dendritic cell (DC) subsets in the Peyer’s patches and mesenteric lymph nodes. Cells were selected based on their characteristic forward and side scatter properties. Next, live cells (Zombie Green^{-}) were selected, whereas dead cells (Zombie Green^{+}), B cells (CD19^{+}/B220^{+}) and natural killer cells (Nkp46^{+}) were excluded. Subsequently, antigen presenting cells were selected (MHCII^{+}). Within this selection, the cell population containing the DCs (CD64^{-}) were selected whereas macrophages (MHCII^{+}CD64^{+}) were excluded. Next, DCs were selected (MHCII^{+}CD64^{-}CD11c^{+}). Within the DC population we identified three subsets: CD103^{+}CD11b^{-} DCs, CD103^{+}CD11b^{+} DCs and CD10^{-}CD11b^{+} DCs. The gates of MHCII, CD64 and CD11b were set using “Fluorescence Minus One” controls.

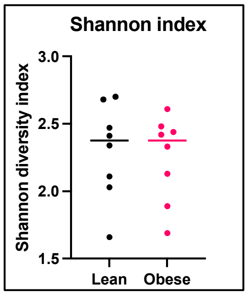

Supplementary Figure S4: Shannon diversity index in the feces of lean and obese mice measured on pregnancy day 12. Data are presented as individual values and median. Mann-Whitney-U-test. Lean mice: n = 8. Obese mice: n = 8.

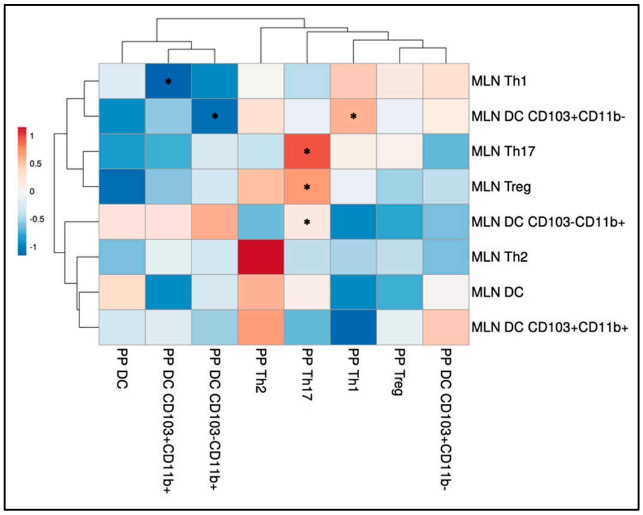

Supplementary Figure S5: Immune cells in the Peyer’s patches (PPs) correlate with immune cells in the mesenteric lymph nodes (MLNs). Heatmap of Spearman’s correlation coefficients after individual correlation of intestinal immune cell populations in the PPs (x-axis) and MLNs (y-axis) at day 12 of pregnancy. Lean mice: $n = 8$. Obese mice: $n = 8$. * $p < 0.05$. Several significant correlations were found between immune cell subsets in the PPs and MLNs. $CD103^+CD11b^+$ and $CD103^-CD11b^+$ DCs in the PPs respectively correlated negatively with Th1 cells and $CD103^+CD11b^-$ DCs in the MLNs. The percentage of Th17 cells in the PP correlated positively with percentages of Th17 cells, Treg cells and $CD103^-CD11b^+$ DCs in the MLNs. Th1 cells in the PPs correlated positively with $CD103^+CD11b^-$ DCs in the MLNs.

Supplementary Table S1. Immune cell subsets stained in this study, including their main function

**Cell type**	**Marker (mouse)**	**Main function**
T helper 1 (Th1)	Tbet, IFN-γ	Promoting cell-mediated immunity [1]
T helper 2 (Th2)	GATA-3, IL-4	Promoting humoral immunity [1]
T helper 17 (Th17)	RORγT, IL-17A	Facilitating host defense against pathogen infection [1]
Regulatory T (Treg)	FoxP3, IL-10	Maintaining immune tolerance and suppressing excessive immune responses [1]
Monocytes (general)	$CD11b^{+}$, $Ly6G^{-}$, $CD115^{+}$	Phagocytosis, antigen presentation, cytokine production, tissue repair and remodeling [2]
Classical monocytes	$CD43^{low}$, $Ly6C^{high}$	Phagocytosis [2]
Intermediate monocytes	$CD43^{+}$, $Ly6C^{int}$	Antigen presentation and production of pro-inflammatory cytokines [2]
Non-classical monocytes	$CD43^{+}$, $Ly6C^{low}$	Tissue repair and remodeling [2]
Dendritic cells (general)	$MHCII^{+}$, $CD64^{-}$, $CD11c^{+}$	Antigen presentation, immune activation, tolerance induction, pathogen detection
$CD103^{+}CD11b^{-}$ dendritic cells	$CD103^{+}$, $CD11b^{-}$	Cross-presentation, immune surveillance, migration to lymph nodes, immune cell activation/differentiation (especially Th1) [3]
$CD103^{+}CD11b^{+}$ dendritic cells	$CD103^{+}$, $CD11b^{+}$	Antigen presentation, maintaining mucosal immunity and barrier integrity, immune cell activation/differentiation (especially Th17), migration to lymph nodes [3,4]
$CD103^{-}CD11b^{+}$ dendritic cells	$CD103^{-}$, $CD11b^{+}$	Antigen presentation, induction of pro-inflammatory responses, immune cell activation/differentiation (especially Th17), facilitating host defense against pathogen infection [3]

References:

1. Saravia, J.; Chapman, N.M.; Chi, H. Helper T cell differentiation. *Cell Mol Immunol* **2019**, *16*, 634-643, doi:10.1038/s41423-019-0220-6.
2. Kapellos, T.S.; Bonaguro, L.; Gemund, I.; Reusch, N.; Saglam, A.; Hinkley, E.R.; Schultze, J.L. Human Monocyte Subsets and Phenotypes in Major Chronic Inflammatory Diseases. *Front Immunol* **2019**, *10*, 2035, doi:10.3389/fimmu.2019.02035.
3. Joeris, T.; Muller-Luda, K.; Agace, W.W.; Mowat, A.M. Diversity and functions of intestinal mononuclear phagocytes. *Mucosal Immunol* **2017**, *10*, 845-864, doi:10.1038/mi.2017.22.
4. Persson, E.K.; Uronen-Hansson, H.; Semmrich, M.; Rivollier, A.; Hagerbrand, K.; Marsal, J.; Gudjonsson, S.; Hakansson, U.; Reizis, B.; Kotarsky, K.; et al. IRF4 transcription-factor-dependent CD103(+)CD11b(+) dendritic cells drive mucosal T helper 17 cell differentiation. *Immunity* **2013**, *38*, 958-969, doi:10.1016/j.immuni.2013.03.009.

Supplementary Table S2. Antibody mix for T helper cell staining

Antibody	Clone	Fluorochrome	Manufacturer	Cat.	Mix	Dilution
anti-CD3	17A2	BV605	Biolegend	100237	Extracellular	1:25
anti-CD4	GK1.5	PerCp-Cy5.5	Biolegend	100434	Extracellular	1:75
anti-CD8	53-6.7	PE-Cy7	Biolegend	100722	Extracellular	1:200
anti-Tbet	4B10	BV421	Biolegend	644816	Intracellular	1:10
anti-Rorγt	B2D	PE	Invitrogen	12-6981-82	Intracellular	1:100
anti-Gata3	L50-823	AF647	BD Biosciences	560068	Intracellular	1:100
anti-FoxP3	FJK-16s	FITC	Invitrogen	11-5773-82	Intracellular	1:50

Supplementary Table S3. Antibody mix for dendritic cell staining

Antibody	Clone	Fluorochrome	Manufacturer	Cat.	Mix	Dilution
anti-MHCII	M5/114.15.2	PerCp-Cy5.5	Biolegend	107626	Extracellular	1:200
anti-CD11c	HL3	APC	BD Biosciences	550261	Extracellular	1:50
anti-CD64	X54-5/7.1	PE-Cy7	Biolegend	139314	Extracellular	1:25
anti-CD19	6D5	FITC	Biolegend	115506	Extracellular	1:25
anti-B220	RA3-6B2	FITC	Biolegend	103206	Extracellular	1:25
anti-Nkp46	29A1.4	FITC	Biolegend	137606	Extracellular	1:25
anti-CD103	2E7	BV421	Biolegend	121422	Extracellular	1:20
anti-CD11b	M1/70	PE	Biolegend	101208	Extracellular	1:50

Supplementary Table S4. Antibody mix for cytokine producing splenic T helper cell staining

Antibody	Clone	Fluorochrome	Manufacturer	Cat.	Mix	Dilution
anti-CD3	17A2	BV605	Biolegend	100237	Extracellular	1:25
anti-CD4	RM4-5	V500	BD Biosciences	560782	Extracellular	1:100
anti-CD8	53-6.7	PerCp-Cy5.5	Biolegend	100734	Extracellular	1:50
anti-IFNγ	XMG1.2	PE	Biolegend	505808	Intracellular	1:500
anti-IL-4	BVD6-24G2	FITC	Invitrogen	11-7042-82	Intracellular	1:400
anti-IL-17A	TC11-18H10.1	APC	Biolegend	506916	Intracellular	1:500
anti-IL-10	JES5-16E3	BV421	Biolegend	505022	Intracellular	1:250

Supplementary Table S5. Antibody mix for monocyte staining

Antibody	Clone	Fluorochrome	Manufacturer	Cat.	Mix	Dilution
anti-CD11b	M1/70	PE	Biolegend	101208	Extracellular	1:50
anti-Ly6G	1A8	BV605	BD Biosciences	563005	Extracellular	1:25
anti-CD115	AFS98	PE-Cy7	Biolegend	135525	Extracellular	1:60
anti-CD43	S11	APC	Biolegend	143208	Extracellular	1:100
anti-Ly6C	HK1.4	AF488	Biolegend	128022	Extracellular	1:200
anti-CD80	16-10A1	PB	Biolegend	104724	Extracellular	1:25
